# Comparative efficacy and safety of etoposide plus PEG-rhG-CSF versus etoposide plus G-CSF for haematopoietic stem cell mobilisation in patients with multiple myeloma and lymphoma

**DOI:** 10.3389/fonc.2026.1879386

**Published:** 2026-06-17

**Authors:** Yiming Zhao, Lixia Liu, Wei Qian, Jianjun Li, Liang Xia

**Affiliations:** 1 Department of Hematology, The First Affiliated Hospital of Anhui Medical University, Hefei, China; 2 Department of Hematology, Anhui Public Health Clinical Center, Hefei, China

**Keywords:** autologous haematopoietic stem cell transplantation, etoposide, lymphoma, mecapegfilgrastim, multiple myeloma, recombinant human granulocyte colony-stimulating factor

## Abstract

**Introduction:**

Efficient and safe mobilisation of haematopoietic stem cells is essential for successful autologous haematopoietic stem cell transplantation (ASCT). Although pegylated recombinant human granulocyte colony stimulating factor, (PEG-rhG-CSF) is widely used for chemotherapy-induced neutropenia, comparative data on its use with high-dose etoposide (VP-16) for mobilisation remain scarce. In particular, direct comparisons between mecapegfilgrastim and recombinant human G-CSF (rhG-CSF) in this setting are lacking.

**Methods:**

In this retrospective study, we analysed the efficacy and safety of high-dose VP-16 (1.6 g/m^2^) combined with PEG-rhG-CSF (100 μg/kg) versus high-dose VP-16 (1.6 g/m^2^) combined with rhG-CSF (10 μg/kg/d) for stem cell mobilisation.

**Results:**

Seventy-one patients were included: 32 received VP-16+PEG-rhG-CSF and 39 received VP-16+rhG-CSF. Baseline characteristics were comparable. Mobilisation success exceeded 90% in both groups. However, the PEG-rhG-CSF group achieved a significantly higher total CD34^+^ cell yield, a greater proportion of optimal mobilisers, and a higher first-day collection success rate. Fewer number of apheresis and a shorter mobilisation duration were observed in this group. The incidence of febrile infection was significantly lower with PEG-rhG-CSF, whereas platelet transfusion requirements and engraftment kinetics were similar between groups.

**Conclusions:**

High-dose VP-16 combined with PEG-rhG-CSF demonstrated superior efficacy, safety, and procedural efficiency compared with high-dose VP-16 plus rhG-CSF. These findings suggest that this regimen represents a promising and potentially preferable alternative for haematopoietic stem cell mobilisation in patients with multiple myeloma or lymphoma.

## Introduction

Autologous haematopoietic stem cell transplantation (ASCT) is one of the most important therapeutic approaches for patients with lymphoma and multiple myeloma. At present, mobilised peripheral blood stem cells have largely replaced bone marrow as the main source of stem cells for ASCT because they are rich in CD34^+^ haematopoietic stem cells, are easier to collect, and promote rapid haematopoietic reconstitution (1, 2). The success of ASCT depends on the collection of an adequate number of CD34^+^ haematopoietic stem cells; the minimum required threshold is generally ≥2 × 10^6^ cells/kg, whereas ≥5 × 10^6^ cells/kg is considered the optimal infusion dose (3). However, there are significant interindividual differences in the number of peripheral blood stem cells. Therefore, an ideal mobilisation regimen should efficiently and reliably obtain sufficient stem cells while ensuring safety and tolerability.

Multiple myeloma accounts for approximately 1% of all malignancies and 10% of haematological cancers (4). Current treatment strategies for this disease mainly include high-dose chemotherapy combined with ASCT, immunomodulatory agents, proteasome inhibitors, and monoclonal antibodies. Among these approaches, ASCT remains the standard treatment for eligible patients (5). Studies have demonstrated that, compared with conventional chemotherapy, ASCT can significantly improve event-free survival and median overall survival in patients with multiple myeloma (6). In addition, ASCT is also applicable to various lymphoma subtypes, including diffuse large B-cell lymphoma, mantle cell lymphoma, and peripheral T-cell lymphoma (7, 8).

Granulocyte colony-stimulating factor (G-CSF) is the standard agent currently used for peripheral blood haematopoietic stem cell mobilisation and may be administered alone or in combination with chemotherapy (9). Commonly used recombinant human G-CSF preparations in clinical practice, such as filgrastim, lenograstim, and mecapegfilgrastim, can effectively reduce the risk of adverse events, including febrile neutropenia (10). However, mobilisation with G-CSF alone may result in insufficient CD34^+^ cell collection or even mobilisation failure. Known risk factors include advanced age, multiple prior lines of chemotherapy, and diabetes mellitus (11, 12). Mobilisation failure directly precludes the implementation of ASCT as a potentially curative treatment and may have a serious adverse impact on patient prognosis (7).

To optimise mobilisation efficiency, several strategies have been developed in clinical practice, including cytokine mobilisation alone, chemotherapy combined with cytokines, and G-CSF in combination with the CXCR4 antagonist plerixafor (13). Among these approaches, etoposide (VP-16) combined with G-CSF has demonstrated a high mobilisation success rate and increased CD34^+^ cell yield, with good tolerability (14, 15). However, the high cost of plerixafor limits its broader clinical applicability.

Mecapegfilgrastim, a pegylated recombinant human granulocyte colony stimulating factor (PEG-rhG-CSF), is a representative long-acting formulation with a mechanism of action similar to that of recombinant human granulocyte colony-stimulating factor (rhG-CSF), but a prolonged half-life, allowing for single-dose administration and improved patient convenience (16, 17). In 2018, it was approved by the China National Medical Products Administration for reducing the risk of chemotherapy-related infections in patients with non-myeloid malignancies (16). Previous studies have preliminarily demonstrated its potential application in haematopoietic stem cell mobilisation (18). Compared with short-acting rhG-CSF, which requires daily administration, PEG-rhG-CSF has also shown greater cost-effectiveness in pharmacoeconomic evaluations (19). Clinical studies have indicated that PEG-rhG-CSF achieves at least comparable mobilisation efficacy to rhG-CSF, with greater convenience and no serious adverse events reported (20). Although the National Comprehensive Cancer Network (NCCN) Guidelines (Version 1, 2022) recommend pegfilgrastim for autologous donor stem cell mobilisation and state that its biosimilars may serve as alternative options, mecapegfilgrastim, as a biosimilar of pegfilgrastim, has been developed and marketed in China. However, clinical data regarding its use in haematopoietic stem cell mobilisation remain limited (21).

In light of the above, this study aimed to comprehensively compare the efficacy of high-dose VP-16 combined with rhG-CSF versus high-dose VP-16 combined with PEG-rhG-CSF for haematopoietic stem cell mobilisation in patients with lymphoma and multiple myeloma. By clarifying the relative efficacy and safety profiles of these two regimens, our findings may provide evidence to inform the selection of an optimal mobilisation strategy and thereby improve the overall success rate of ASCT in patients with lymphoma and multiple myeloma.

## Methods

### Patients

**Figure 1:**
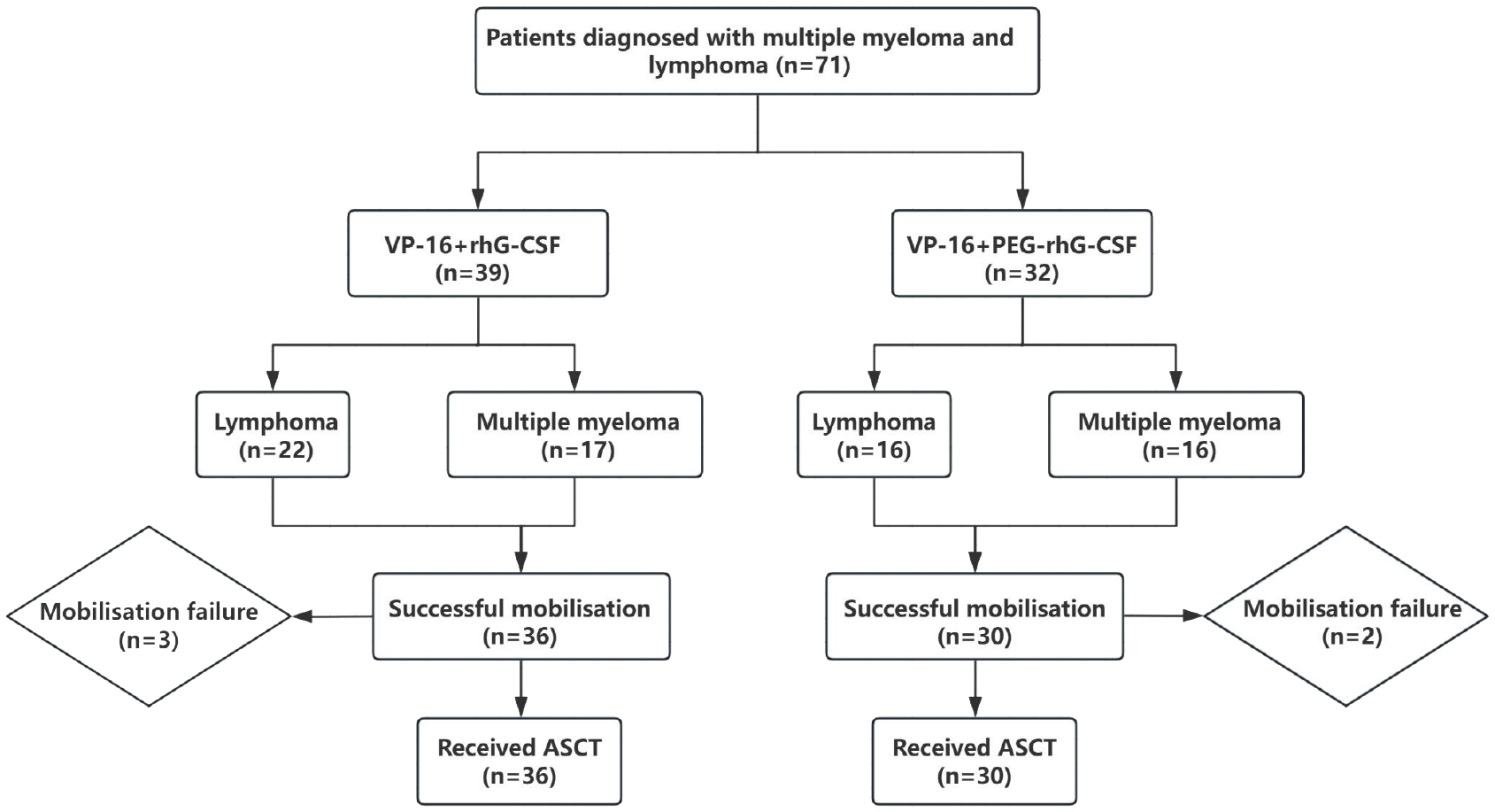
Study flowchart.

This study included patients who underwent autologous peripheral blood haematopoietic stem cell collection in the Department of Haematology, North District of the First Affiliated Hospital of Anhui Medical University, between January 2020 and December 2025. The study flowchart is shown in **Figure 1**.

The inclusion criteria were as follows: (1) age between 18 and 70 years; (2) diagnosis of multiple myeloma or lymphoma; (3) achievement of complete or partial remission after treatment; (4) a clear indication for autologous peripheral blood haematopoietic stem cell transplantation; and (5) an Eastern Cooperative Oncology Group (ECOG) performance status score of 0–2.

The exclusion criteria were as follows: (1) severe cardiopulmonary dysfunction or other serious comorbidities that might increase treatment-related risk; (2) active infection diagnosed during the screening period; (3) pregnancy or lactation; and (4) prior receipt of allogeneic stem cell transplantation.

After study initiation, data were collected from the medical records system, including baseline demographic characteristics, medical history, disease type, number of prior chemotherapy cycles, type of mobilisation regimen, adverse events, time from mobilisation to collection, number of apheresis sessions, CD34^+^ cell yield, and treatment-related adverse events.

This study was conducted in accordance with the Declaration of Helsinki and was approved by the Ethics Committee of the North District of the First Affiliated Hospital of Anhui Medical University.

### Mobilisation regimens

VP-16+rhG-CSF group: Patients received VP-16 injection at a dose of 1.6 g/m² administered as a continuous intravenous infusion over 10 hours on Day 1. During the infusion, methylprednisolone 40 mg was administered intravenously at 0, 4, and 8 hours (three doses in total) to prevent VP-16-related adverse reactions. Complete blood counts were closely monitored. When the absolute neutrophil count (ANC) decreased to < 0.5 × 10^9^/L, rhG-CSF (10 μg/kg/day; Kyowa Kirin Co., Ltd.) was administered subcutaneously. Peripheral blood counts and CD34^+^ cell levels were dynamically monitored, and stem cell collection was initiated when the peripheral blood CD34^+^ cell count exceeded 20/μL.

VP-16+PEG-rhG-CSF group: Patients received VP-16 injection at a dose of 1.6 g/m² administered as a continuous intravenous infusion over 10 hours on Day 1. During the infusion, methylprednisolone 40 mg was administered intravenously at 0, 4, and 8 hours (three doses in total) to prevent VP-16-related adverse reactions. On Day 5, mecapegfilgrastim at a dose of 100 μg/kg (Jiangsu Hengrui Pharmaceuticals Co., Ltd.) was administered subcutaneously. Peripheral blood counts and CD34^+^ cell levels were dynamically monitored, and stem cell collection was initiated when the peripheral blood CD34^+^ cell count exceeded 20/μL.

### Collection and cryopreservation of peripheral blood haematopoietic stem cells

After initiation of mobilisation, complete blood counts were routinely monitored. When the white blood cell (WBC) count recovered to ≥ 1.0 × 10^9^/L, peripheral blood CD34^+^ cell counts and nucleated cell counts were measured using flow cytometry. When the CD34^+^ cell level exceeded 20/μL, haematopoietic stem cell collection was performed using a COM.TEC cell separator (Fresenius Kabi). The Auto MNC (stem cell) programme was selected, and a P1Ya disposable kit was used. Circulating blood volume, number of cycles, and target product volume were set according to standard procedures before collection. After the first apheresis session, the number of CD34^+^ cells/kg and mononuclear cells (MNC)/kg was calculated based on the patient’s body weight on that day. Collection was considered successful if the total number of CD34^+^ cells was ≥ 2.0 × 10^6^/kg. If this threshold was not reached, collection was continued on the following day until the cumulative CD34^+^ cell count reached ≥ 2.0 × 10^6^/kg. Mobilisation failure was defined as a cumulative CD34^+^ cell count of < 2.0 × 10^6^/kg after three consecutive days of collection. The absolute CD34^+^ cell count in the collected product was determined using a flow cytometric bead-based method with a Navios flow cytometer (Beckman Coulter). The number of collection sessions was evaluated according to the absolute CD34^+^ cell yield. Following successful collection, stem cells were aliquoted in cryopreservation solution (Nanjing Sansheng Pharmaceutical Co., Ltd.), carefully documented and labelled, and stored at −80 °C.

Mobilisation definitions: (1) mobilisation success: CD34^+^ cell count per body weight ≥ 2 × 10^6^/kg; (2) optimal mobilisation: CD34^+^ cell count ≥ 5 × 10^6^/kg; (3) mobilisation failure: CD34^+^ cell count per body weight < 2 × 10^6^/kg.

### Statistical analysis

Statistical analyses were performed using SPSS version 26.0 and GraphPad Prism version 9.4.1. Following assessment of normality, most variables were found to be non-normally distributed. Continuous variables are presented as mean ± standard deviation for normally distributed data and as median (interquartile range, IQR) for non-normally distributed data. Between-group comparisons were conducted using the independent-samples *t*-test or the Mann–Whitney *U* test, as appropriate. Categorical variables are expressed as number (percentage) and were compared using the *χ²* test or Fisher’s exact test. A two-sided *P*-value < 0.05 was considered statistically significant.

## Results

### Patient clinical characteristics

A total of 71 patients were included in this study, with a median age of 54 (48, 58) years. Among them, 39 patients received the VP-16+rhG-CSF mobilisation regimen, and 32 patients received the VP-16+PEG-rhG-CSF regimen. There were no statistically significant differences between the two groups in terms of age, sex, body mass index, disease type (lymphoma or multiple myeloma), presence of diabetes mellitus, disease status (partial response or complete response), number of prior chemotherapy cycles, or platelet count at the time of collection (all *P* > 0.05), as shown in **Table 1**.

**Table 1:** Baseline demographic and clinical characteristics of the patients.

Clinical characteristic	VP-16+rhG-CSF ( n =39 )	VP-16+PEG-rhG-CSF ( n =32 )	*t/z/χ2*	*P*-value
Age	53.00 (48.00, 58.00)	54.00 (45.50, 60.00)	-0.040	0.968
Gender				
Male	23 (54.80%)	19 (45.20%)	0.001	0.973
Female	16 (55.20%)	13 (44.80%)		
BMI, kg/m2	24.71±3.16	24.36±2.79	0.493	0.624
Diseases				
Multiple myeloma	17 (43.60%)	16 (50.00%)	0.290	0.590
Lymphoma	22 (56.40%)	16 (50.00%)		
Diabetes				
Yes	6 (15.40%)	10 (31.20%)	2.534	0.111
No	33 (84.60%)	22 (68.80%)		
Status of disease at collection				
CR	18 (46.20%)	11 (34.40%)	1.009	0.315
PR	21 (53.80%)	21 (65.60%)		
Lines of prior chemotherapy				
<6	21 (53.80%)	21 (65.60%)	1.009	0.315
≥6	18 (46.20%)	11 (34.40%)		
Platelet at the time of collection	63.00 (39.00, 85.00)	45.00 (36.50, 64.25)	-1.578	0.115

BMI, body mass index; CR, complete response; PEG-rhG-CSF, pegylated recombinant human granulocyte colony stimulating factor; PR, partial response; rhG-CSF, recombinant human granulocyte colony-stimulating factor; VP-16, etoposide.

### Comparison of efficacy between the two mobilisation regimens

**Figure 2:**
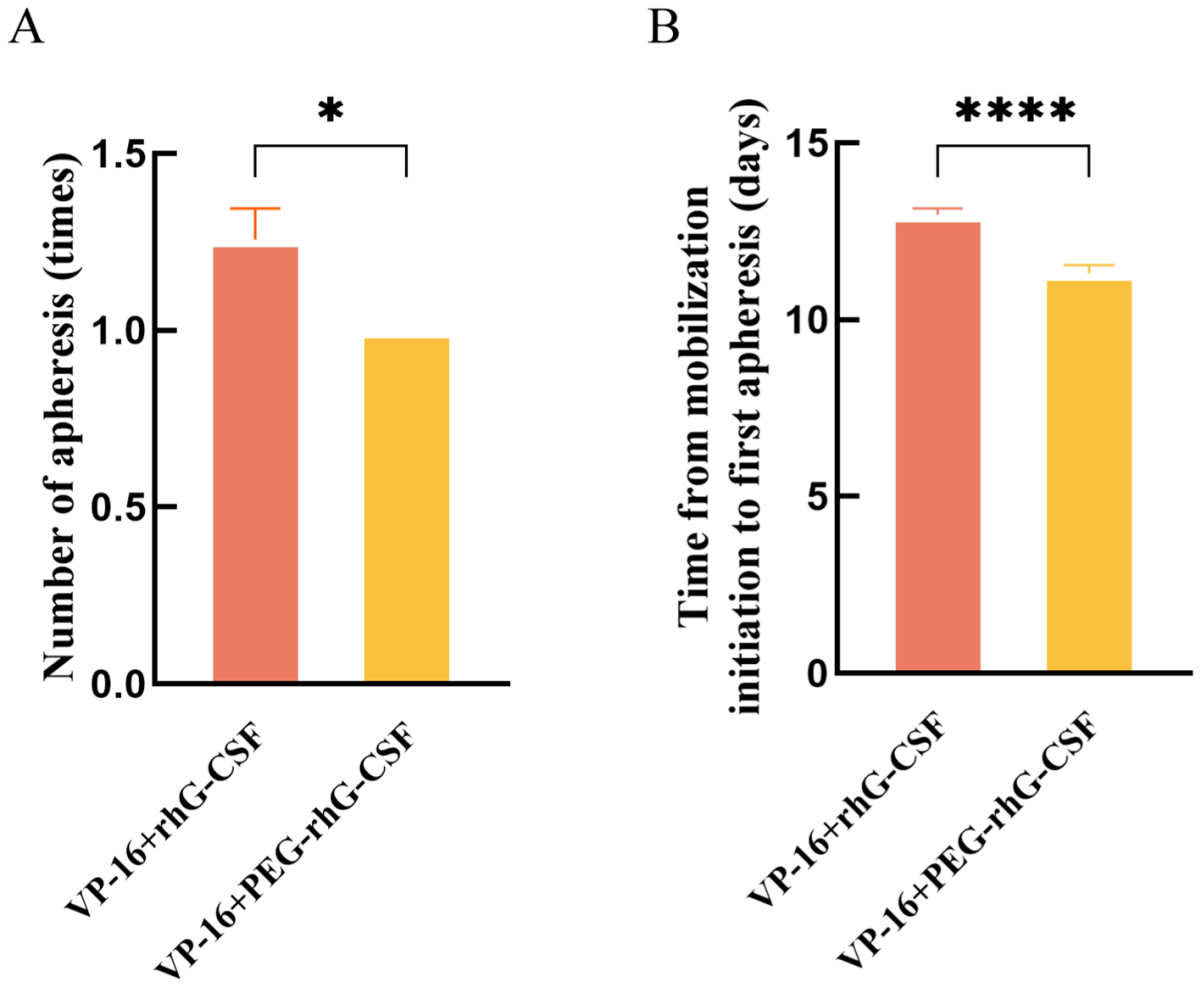
Comparison of the two mobilisation regimens. **(A)** Number of apheresis sessions; **(B)** Time from mobilisation to collection. PEG-rhG-CSF, pegylated recombinant human granulocyte colony stimulating factor; rhG-CSF, recombinant human granulocyte colony-stimulating factor; VP-16, etoposide. *p<0.05, ****p<0.0001.

Among the 71 patients, 66 achieved successful mobilisation. The mobilisation success rate was 93.8% in the VP-16+PEG-rhG-CSF group and 92.3% in the VP-16+rhG-CSF group, with no statistically significant difference between the groups (*P* > 0.05). The median CD34^+^ cell yield was significantly higher in the VP-16+PEG-rhG-CSF group than in the VP-16+rhG-CSF group 6.56 (4.00, 11.92) × 10^6^/kg vs 3.60 (2.30, 7.60) × 10^6^/kg. The proportion of patients achieving optimal mobilisation was greater in the VP-16+PEG-rhG-CSF group (65.6%) than in the VP-16+rhG-CSF group (41.0%). The first-day collection success rate was also higher in the VP-16+PEG-rhG-CSF group (93.8% vs 71.8%), as was the proportion of patients achieving optimal mobilisation on the first collection day (65.6% vs 35.9%). These differences were statistically significant (*P* < 0.05) (**Table 2**). In addition, the number of apheresis sessions and the time from mobilisation to collection were both significantly lower in the VP-16+PEG-rhG-CSF group compared with the VP-16+rhG-CSF group (*P* < 0.05) (**Figure 2**). However, there were no statistically significant differences between the two groups in the total mononuclear cell yield or overall mobilisation efficiency (*P* > 0.05) (**Table 2**).

**Table 2:** Comparison of mobilization efficacy in the VP-16+rhG-CSF group and the VP-16+PEG-rhG-CSF group.

Clinical characteristic	VP-16+rhG-CSF ( n =39 )	VP-16+PEG-rhG-CSF ( n =32 )	*z/χ2*	*P*-value
Collection of CD34+cell counts (10^6^/kg)	3.60 (2.30, 7.60)	6.56 (4.00, 11.92)	-2.849	0.004
≥2×10^6^ CD34^+^/kg	36 (92.30%)	30 (93.80%)	0.056	0.813
≥5×10^6^ CD34^+^/kg	16 (41.00%)	21 (65.60%)	4.262	0.039
≥2×10^6^ CD34^+^/kg at the first apheresis	28 (71.80%)	30 (93.80%)	5.665	0.017
≥5×10^6^ CD34^+^/kg at the first apheresis	14 (35.90%)	21 (65.60%)	6.215	0.013
Mobilization efficacy				
Optimal	16 (41.00%)	21 (65.60%)	4.401	0.111
Standard	20 (51.30%)	9 (28.10%)		
Failure	3 (7.70%)	2 (6.30%%)		
Collection of MNC counts (10^8^/kg)	3.36 (2.75, 5.03)	3.63 (2.26, 5.69)	-0.237	0.813

MNC, mononuclear; PEG-rhG-CSF, pegylated recombinant human granulocyte colony stimulating factor; rhG-CSF, recombinant human granulocyte colony-stimulating factor; VP-16, etoposide.

### Comparison of adverse events and post-ASCT haematological recovery between the two regimens

In the VP-16+rhG-CSF group, 20 patients (51.3%) developed febrile infection, which was significantly higher than the 9 patients (28.1%) in the VP-16+PEG-rhG-CSF group (*P* < 0.05). Platelet transfusion was required in 9 patients (23.1%) in the VP-16+rhG-CSF group, compared with 4 patients (12.5%) in the VP-16+PEG-rhG-CSF group; however, this difference was not statistically significant (*P* > 0.05) (**Table 3**).

**Table 3:** Comparison of adverse reaction in the VP-16+rhG-CSF group and the VP-16+PEG-rhG-CSF group.

Clinical characteristic	VP-16+rhG-CSF ( n =39 )	VP-16+PEG-rhG-CSF ( n =32 )	*χ2*	*P*-value
Occurrence of infection				
Yes	20 (51.30%)	9 (28.10%)	3.901	0.048
No	19 (48.70%)	23 (71.90%)		
Transfusion of PLT (therapeutic volumes)				
Yes	9 (23.10%)	4 (12.50%)	1.315	0.252
No	30 (76.90%)	28 (87.50%)		

PEG-rhG-CSF, pegylated recombinant human granulocyte colony stimulating factor; rhG-CSF, recombinant human granulocyte colony-stimulating factor; VP-16, etoposide.

**Figure 3:**
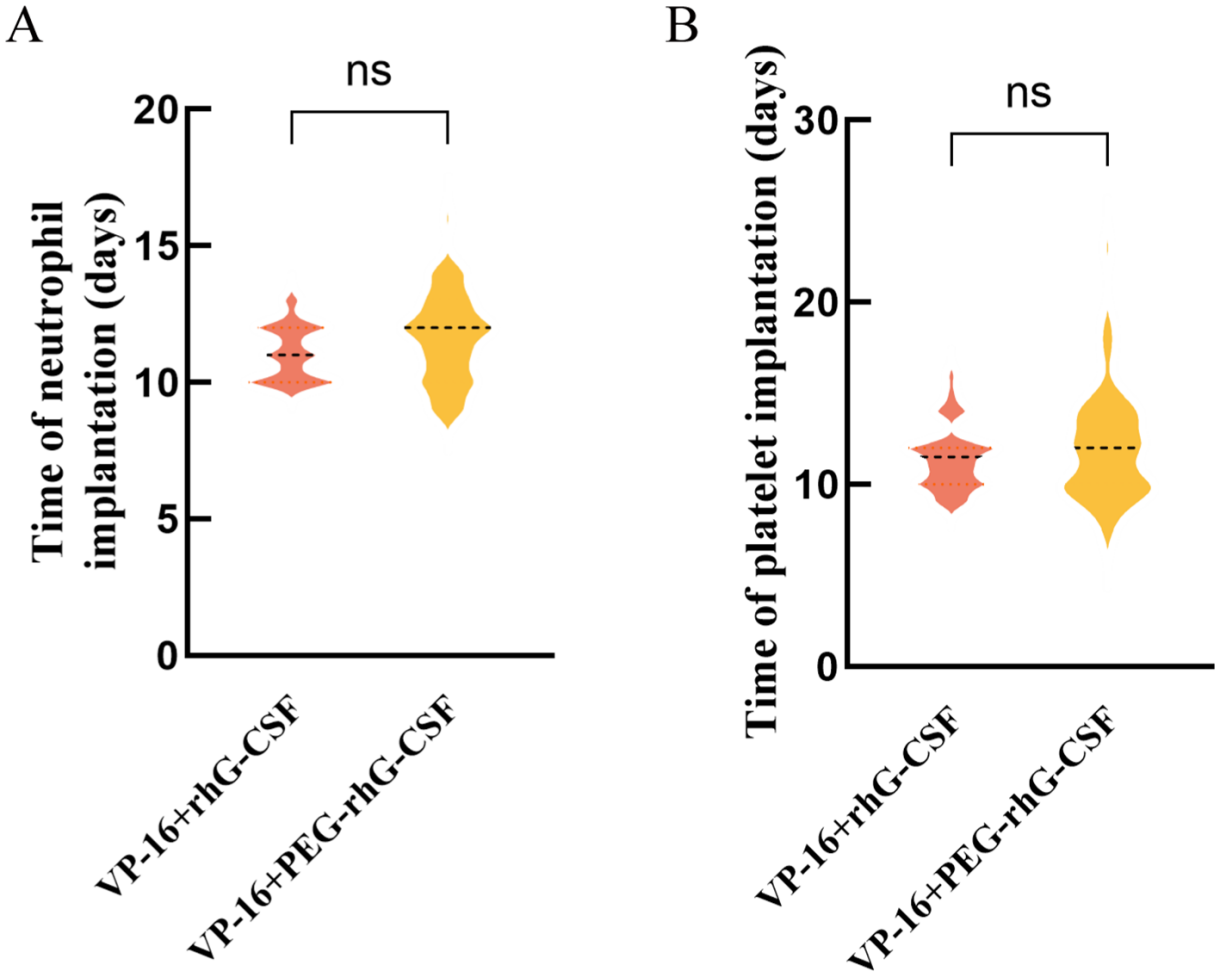
Comparison of neutrophil and platelet engraftment after ASCT between the two groups. **(A)** Comparison of time to neutrophil engraftment; **(B)** Comparison of time to platelet engraftment. PEG-rhG-CSF, pegylated recombinant human granulocyte colony stimulating factor; rhG-CSF, recombinant human granulocyte colony-stimulating factor; VP-16, etoposide.

With regard to transplantation and haematological reconstitution, 36 patients (92.3%) in the VP-16+ rhG-CSF group proceeded to transplantation, compared with 30 patients (93.8%) in the VP-16+PEG-rhG-CSF group; the difference was not statistically significant (*P* > 0.05). Notably, no cases of graft failure occurred in either group. The median time to neutrophil engraftment in the VP-16+rhG-CSF group was 12.00 (10.00, 13.00) days, and the median time to platelet engraftment was 12.00 (10.00, 14.00) days. These were slightly longer than those observed in the VP-16+PEG-rhG-CSF group, in which the median time to neutrophil engraftment was 11.00 (10.00, 12.00) days and the median time to platelet engraftment was 11.50 (10.00, 12.00) days; however, the differences were not statistically significant (*P* > 0.05) (**Figure 3**).

## Discussion

The success of ASCT is highly dependent on a safe and efficient haematopoietic stem cell mobilisation strategy. In recent years, PEG-rhG-CSF has been widely used in the prevention and management of chemotherapy-induced neutropenia (16). However, clinical evidence regarding the use of PEG-rhG-CSF in combination with high-dose VP-16 for stem cell mobilisation remains limited. In particular, direct comparative studies evaluating mobilisation efficiency, safety, and transplantation outcomes between the long-acting formulation mecapegfilgrastim and conventional short-acting rhG-CSF are lacking. Accordingly, the present study is the first to compare high-dose VP-16 combined with mecapegfilgrastim versus high-dose VP-16 combined with short-acting rhG-CSF in terms of mobilisation efficacy, safety, and post-transplant haematopoietic recovery, thereby providing new evidence to inform the optimisation of clinical mobilisation strategies.

The results of this study demonstrated that the overall mobilisation success rate exceeded 90% in both the VP-16+rhG-CSF and VP-16+PEG-rhG-CSF groups, indicating that VP-16 combined with G-CSF-based regimens is a reliable mobilisation strategy for patients with multiple myeloma and lymphoma (14, 22, 23). Although no statistically significant difference was observed in overall mobilisation success between the two groups, the VP-16+PEG-rhG-CSF regimen showed clear advantages across several key indicators reflecting mobilisation quality and clinical efficiency. Patients in the PEG-rhG-CSF group achieved a higher total CD34^+^ cell yield, a greater proportion of optimal mobilisation, and significantly higher rates of first-day collection success and first-day optimal mobilisation compared with the VP-16+rhG-CSF group. These findings suggest that long-acting G-CSF may enhance the stability and efficiency of haematopoietic stem cell mobilisation. This is broadly consistent with previous reports. Earlier studies evaluating chemotherapy combined with PEG-rhG-CSF for stem cell mobilisation in patients with multiple myeloma and lymphoma have reported success rates ranging from 80.6% to 92.1% (21, 24). However, direct comparisons between long-acting and short-acting G-CSF regimens have yielded inconsistent conclusions: some studies suggest that PEG-rhG-CSF is superior to rhG-CSF for mobilisation (21, 25), whereas others report a potential advantage of short-acting G-CSF or comparable efficacy between the two (20, 26). The findings of the present study support the view that, in the context of high-dose VP-16, long-acting G-CSF confers a clear advantage in improving mobilisation quality and efficiency.

From a biological perspective, PEG-rhG-CSF has a longer bioavailability than short-acting rhG-CSF, which may confer an advantage during haematopoietic stem cell mobilisation (26). In addition, the single-dose administration schedule may improve patient adherence. Previous studies have shown that, compared with short-acting G-CSF, PEG-rhG-CSF stimulation results in a higher total nucleated cell count in the first leukapheresis product from donors, and the mobilised grafts may exhibit enhanced immunomodulatory properties (27). Furthermore, peripheral blood stem cells mobilised by PEG-rhG-CSF demonstrate gene expression profiles more characteristic of immature progenitor cells and are associated with mobilisation of a higher proportion of common myeloid progenitors (28).

In addition, the present study demonstrated that the VP-16+PEG-rhG-CSF group required fewer apheresis sessions and a shorter interval from initiation of collection to completion of mobilisation. These findings are of considerable clinical relevance, as they may reduce patient discomfort and potential risks associated with repeated apheresis procedures, while also decreasing healthcare resource utilisation and improving the overall efficiency of the transplantation process. With regard to safety, the incidence of febrile infection was significantly higher in the VP-16+rhG-CSF group than in the VP-16+PEG-rhG-CSF group. Previous studies have likewise shown that chemotherapy combined with PEG-rhG-CSF can significantly reduce the incidence of febrile neutropenia (29). In patients with breast cancer, multiple studies have confirmed that PEG-rhG-CSF is more effective and more convenient than rhG-CSF in preventing neutropenia (30, 31). Similarly, in newly diagnosed acute lymphoblastic leukaemia, PEG-rhG-CSF has been shown to reduce the incidence of post-induction neutropenia without compromising anti-tumour efficacy (32). The lower rate of febrile infection observed in the PEG-rhG-CSF group in our study may be related to these effects.

Finally, there was no statistically significant difference between the two groups in terms of platelet transfusion requirements, suggesting that the two regimens are broadly comparable with respect to platelet-related safety. Regarding transplantation and haematopoietic reconstitution, both groups exhibited high transplantation completion rates, and no cases of secondary graft failure were observed. Although the VP-16+PEG-rhG-CSF group showed slightly shorter times to neutrophil and platelet engraftment, these differences did not reach statistical significance. This indicates that the long-acting formulation can achieve haematopoietic recovery that is at least comparable to that of the short-acting agent, without increasing transplantation-related risks.

Although the present study demonstrated that high-dose VP-16 combined with PEG-rhG-CSF was superior to the short-acting rhG-CSF regimen in terms of mobilisation efficiency and safety, several limitations should be acknowledged. First, this was a single-centre study with a relatively limited sample size, and more detailed stratified analyses according to disease subtype and high-risk populations were not performed. In addition, long-term survival outcomes and cost-effectiveness were not systematically evaluated. Therefore, further multicentre, prospective studies are warranted to validate the clinical value of this regimen across different patient populations.

## Conclusions

In summary, high-dose VP-16 combined with mecapegfilgrastim for haematopoietic stem cell mobilisation maintains a high overall mobilisation success rate while significantly increasing CD34^+^ cell yield and first-day collection efficiency, reducing the number of apheresis sessions, and lowering the risk of mobilisation-related infections, without adversely affecting post-transplant haematopoietic recovery. This regimen demonstrates clear advantages in terms of efficacy, safety, and procedural efficiency, and shows promising potential for broader clinical application. Further validation in patients undergoing ASCT is warranted.

## Data Availability

The raw data supporting the conclusions of this article will be made available by the authors, without undue reservation.
